# Benefits and stressors – Perceived effects of ICT use on employee health and work stress: An exploratory study from Austria and Hong Kong

**DOI:** 10.3402/qhw.v10.28838

**Published:** 2015-10-13

**Authors:** Katharina Ninaus, Sandra Diehl, Ralf Terlutter, Kara Chan, Anqi Huang

**Affiliations:** 1 Department of Marketing and International Management, Alpen-Adria Universitaet Klagenfurt, Klagenfurt, Austria; 2 Department of Media and Communication, Alpen-Adria Universitaet Klagenfurt, Klagenfurt, Austria; 3 Department of Communication Studies, Hong Kong Baptist University, Kowloon, Hong Kong

**Keywords:** Work-related technology use, ICT stressors, ICT benefits, work stress, burnout, employee health

## Abstract

Stress has become a mass phenomenon in the modern workplace. The use of information and communication technologies is beginning to receive greater attention in the context of occupational stress. An exploratory qualitative study was conducted to examine both stressors and benefits resulting from technologies among practitioners in the advertising, public relations, and journalism industry in Hong Kong and Austria. Results suggest that technologies allow instant availability, which facilitates communication processes as well as information exchange. Notably, modern technologies enable employees to organize their work with greater temporal and spatial flexibility, thus creating an opportunity for better balancing work and private life. However, evolving technologies have come with a cost; the pressure to be constantly available via technologies constitutes a major source of stress, increasing the risk of experiencing prolonged work stress and its adverse consequences on employee health and well-being, such as a burnout. Furthermore, findings suggest that availability pressure may be attributed to an inner obligation rather than to an organizational expectation. Hence, making employees aware of their connectivity behaviour may help to diminish the experience of technology-induced work stress and improve and maintain employees’ health and well-being in the long term. Practical implications, limitations, and future research directions are provided.

Stress has become *an epidemic* (Conner, 2014, para. 5) in the modern workplace. The World Health Organization has declared occupational stress as one of the major health threats of the twenty-first century (Adli, 2011). Research agrees that prolonged job stress relates to work and health outcomes, such as poor performance, overall health impairments, or burnout (Kahn & Byosiere, 1992; Ozkan & Ozdevecioğlu, 2013). The latter is *a psychological syndrome in response to chronic interpersonal stressors on the job* (Maslach, Schaufeli, & Leiter, 2001, p. 399) comprising the dimension of exhaustion, a cynical attitude towards one's work and employer, and a diminished sense of professional efficacy (Maslach & Jackson, 1981). Although burnout is not considered an autonomous disease in the International Classification of Diseases, but merely an influencing factor (World Health Organization, 2015), it has become a global concern and has been recognized as a major issue for health care policymakers and a challenge to employees’ health and the performance of organizations (Carod-Artal & Vázquez-Cabrera, 2013; Shirom, 2005). Burnout causes high costs and socio-economic consequences, such as decreased performance, increased turnover intention, productivity losses, long absences, chronic work disability, and early retirement (Ahola et al., 2008; Ahola, Toppinen-Tanner, Huuhtanen, Koskinen, & Väänänen, 2009; Bakker, Demerouti, & Verbeke, 2004; Du Plooy & Roodt, 2010; Iacovides, Fountoulakis, Kaprinis, & Kaprinis, 2003). For instance, according to a current report on absence rates released by the Austrian Institute of Economic Research, psychological illnesses, including burnout, have become the second and leading cause of disability pension for men and women, respectively (Leoni, 2014). Hong Kong has recognized mental health as an important health care challenge (Lam et al., 2014). Experts, however, agree that neither the public nor the private sector in Hong Kong is yet placing sufficient emphasis on mental health (Lee & Lam, 2015; Wai-tong, 2015). Results of the recently conducted “Hong Kong Mental Morbidity Survey” funded by the Food and Health Bureau (FHB, 2014) of the Government of the Hong Kong Special Administrative Region show that 13.3% of the 5700 respondents show a prevalence of mental illnesses. Combined with research that has found a relationship between well-being and work performance, in that managers associated health with being highly efficient and effective at work (Mayer & Boness, 2011), it is increasingly important to continuously expand burnout research and to contribute to the understanding of occupational stress and its consequences on employees’ well-being and performance (Shirom, 2005).

One topic that is beginning to receive greater attention in the context of stress, health, and well-being in an organizational context is the use of information and communication technologies (ICTs) in the workplace. ICTs penetrate virtually all areas of life in modern societies, have become an essential part of both leisure and working time (Chesley, Moen, & Shore, 2003; Day, Scott, & Kelloway, 2010), and provide considerable benefits for employers, workers, and society at large (Mamaghani, 2006; O'Driscoll, Brough, Timms, & Sawang, 2010). However, ICTs may also be an additional source of stress, leading to health impairments (Day, Paquet, Scott, & Hambley, 2012; Derks, Ten Brummelhuis, Zecic, & Bakker, 2012; Harris, Marett, & Harris, 2011; Matusik & Mickel, 2011; Ragu-Nathan, Tarafdar, & Ragu-Nathan, 2008; Thomée, Eklöf, Gustafsson, Nilsson, & Hagberg, 2007). Yet, there remains insufficient research on the benefits and downsides of work-related ICT use, particularly on possible adverse effects of technology on employees’ psychological health and well-being (Day et al., 2010; Derks, Van Mierlo, & Schmitz, 2014; Diaz, Chiaburu, Zimmerman, & Boswell, 2012).

The overall purpose of this research is to qualitatively examine both positive and burdening factors of work-related ICT use among employees in the areas of advertising, public relations, and journalism. Previous research has shown that the media sector is vulnerable to work strain and subsequently to exhaustion and cynicism, two core dimensions of burnout (Montgomery, Peeters, Schaufeli, & den Ouden, 2003). Considering these professional fields’ high involvement with technologies, employees may be particularly prone to experiencing prolonged technology-induced work stress and its adverse outcomes.

## Research objectives

This research aims to examine specific stressors and benefits resulting from work-related technology use from an employee's perspective in the areas of advertising, public relations, and journalism. It analyses how employees can benefit from technologies in their communication processes, and how technologies might place additional pressure on employees by, for example, increasing response expectations. This research could provide valuable information on the promotion of employee health in the concerned fields and may provide a suitable basis for developing interventions and preventative measures on an organizational level, which could help to diminish technology-induced work stress, as well as strengthen technology-related benefits. These measures may further contribute to the decrease of occupational stress and its adverse consequences and to the increase of the well-being and satisfaction of employees.

### Country selection

In pursuing these objectives, the research focuses on employees working in Austria and Hong Kong. Although at first glance the two countries appear very different, work stress and workplace health promotion are currently very high on the political agenda in both countries. Hong Kong, for instance, has always had some of the world's longest working hours (Ho, 2006), which had been found to result in health problems and staff turnover (Welford, 2008). The Hong Kong government recognized this and in July 2006 replaced the five and a half day work week with a 5-day work week in the public sectors. A survey among 1027 employees in Hong Kong found that this policy was effective in reducing turnover intention and increasing job satisfaction (Welford, 2008). Long working hours have been found to be the greatest obstacle for employees in Hong Kong in achieving work-life balance which, in turn, was found to result in prolonged fatigue and extreme tiredness (Community Business, 2010). Although the latest report on work-life balance in Hong Kong (Community Business, 2014) highlighted that companies have become increasingly aware of the importance of offering improvements to the working life, many companies in Hong Kong still seem reluctant to change their culture to be more conducive to the development of work-life balance.

In Austria, it is legally mandated that health includes both physical and psychological health. Since the amendment of the Austrian Occupational Health and Safety Act in January 2013, employers are legally obligated to evaluate mental strain in the workplace and to protect employees’ mental health (Ministry for Social Affairs, 2013). Moreover, recent reports by the Austrian Institute of Economic Research (Biffl, Faustmann, Gabriel, Leoni, Mayrhuber, & Rückert, 2012; Leoni, 2014) reveal that sick leave due to work-related mental strains lasts an average of 37 days, which is considerably above the overall average of 12.8 sick leave days per employee. Sick leaves due to work-related mental stress last longer and the medical costs combined with the employers’ expenses amount to 3.3 billion Euros annually (Leoni, 2012; Leoni, 2014). The Austrian Economic Chamber estimates the total costs for the overall Austrian economy due to mental illnesses amount to 7 billion Euros per year (Schuster, 2013).

## Theoretical foundation

In light of the growing interest in positive psychology (Seligman & Csikszentmihalyi, 2000), this research is focusing not only on ICT as an occupational stressor but also on its positive features. It aims to develop ideas for interventions to prevent diseases and promote employee well-being, fostering the idea that health is more than the absence of illness (Slade, 2010). Against this backdrop, the study draws on the Job Demands-Resources Model (JD-R model) (Demerouti, Bakker, Nachreiner, & Schaufeli, 2001), a work stress model that explains how various aspects of the working environment may have positive and negative health and work outcomes (Bakker & Demerouti, 2007). The premise of the model is that regardless of the type of occupation, working conditions can be divided into job demands and job resources. Job demands are physical, psychological, social, or organizational aspects of a job that may require sustained physical and/or psychological effort and are associated with certain physiological and/or psychological costs. Job resources indicate physical, psychological, social, or organizational aspects of a job that may reduce job demands and associated physiological and psychological costs; may be instrumental in achieving work goals; and may promote personal growth, learning, and development (Bakker & Demerouti, 2007; Demerouti et al., 2001).

The JD-R model proposes that chronic and high job demands may become stressors when high effort is required to meet them and may consequently exhaust employees’ resources and cause health impairments. Job resources have a motivational potential and may lead to positive outcomes, such as high work engagement (Bakker & Demerouti, 2007; Gan & Gan, 2014). Regarding burnout in particular, the model assumes that the risk for burnout is highest in working environments where job demands are high and job resources are limited (Demerouti et al., 2001). Accordingly, Bakker, Demerouti, and Euwema (2005) found that high job demands and low job resources lead to higher levels of exhaustion and cynicism. Similarly, Hu, Schaufeli, and Taris (2011) found that high job demands and low job resources lead to more burnout and lower work engagement. In working environments where adequate job resources are available, they may act as a buffer against the negative impact of job demands on employee health and job strain (Bakker & Demerouti, 2007), as evidenced in previous studies (e.g., Bakker et al., 2005; Hu et al., 2011; Xanthopoulou et al., 2007). In fact, it was found that job resources are especially relevant under conditions of high stress (Bakker, Hakanen, Demerouti, & Xanthopoulou, 2007). These findings support the importance of job resources in employee health promotion.

Although originally not considered in the JD-R model, scholars agree that ICTs may also be divided into demands and resources (Patel, Ryoo, & Kettinger, 2012). This is because ICTs may provide potential benefits, but also place additional demands on employees. The ability to be accessible via wireless mobile devices independent of location and time may make flexible working structures increasingly feasible, allowing employees to benefit from increased work time and place flexibility (Cousins & Robey, 2015; Diaz et al., 2012; Jarvenpaa & Lang, 2005). Yet, constant accessibility might also cause permanent interruptions such as phone calls, text messages, or emails. Sellberg and Susi (2014) noted in their observational study that constant interruptions through ICTs scatter a task throughout the day, forcing employees to work faster and longer to meet deadlines. Accessibility regardless of location and time may also promote compulsive checking of missed calls, text messages, or emails (Lee, Chang, Lin, & Cheng, 2014), thus creating expectations for quicker responses and fostering an always-on mentality (Mazmanian, Yates, & Orlikowski, 2006; Park, Fritz, & Jex, 2011).

## Method

### Methodological approach

The study employed an interpretive approach (Neuman, 2003) using qualitative research methodology. Twenty-five individuals were guided, and semi-structured interviews (Eriksson & Kovalainen, 2008) were conducted with 13 participants from Hong Kong and 12 participants from Austria. Such a small number of respondents are characteristic for qualitative research (VanderStoep & Johnston, 2009). Interviews were analysed by performing a computer-supported qualitative content analysis (Mayring, 2000) with the help of QSR NVivo, a qualitative data analysis computer software. Qualitative research is valuable in analysing the field of occupational stress because qualitative methods may identify important aspects of work stress that research has so far overlooked, may allow researchers to gain unconstrained insights into personal work and stress experiences, and may create a basis for hypotheses or instrument development in quantitative research (Schonfeld & Farrell, 2010; Silverman, 2005).

### Participants

Interview participants were recruited through personal networks using purposive sampling, a non-probability sampling technique (Berg & Lune, 2012; Marshall, 1996; Mason, 2002). The criterion for selection was that all participants have common characteristics in terms of industry affiliation and work-related technology use. Based on the aim of the study, included individuals work in the advertising, public relations, or journalism industry and are active users of ICTs in their everyday work life. Participants’ demographic and work profiles are summarized in Table I.

**Table I:** Participants’ demographic and work profiles.

	Sex	Age	Marital status	Job experience	Education	Industry
Hong Kong	Female	21–25	Single	7 months	Master degree	Advertising (agency)
participants	Female	21–25	Single	1.5 years	Master degree	PR (in-house)
	Female	31–35	Single	6 years	Master degree	PR (agency)
	Female	31–35	Single	11 years	Master degree	PR (agency)
	Female	36–40	Married with no child	15 years	First degree	PR (in-house)
	Male	21–25	Single	1.5 years	First degree	Journalism (news magazine)
	Female	41–45	Married with no child	20 years	High school	PR (agency)
	Male	21–25	Single	2.5 years	First degree	Advertising (agency)
	Female	21–25	Single	2.5 years	First degree	Journalism (broadcast)
	Male	26–30	Single	3.5 years	First degree	Advertising (agency)
	Male	26–30	Single	4 years	First degree	Advertising (agency)
	Female	26–30	Single	4 years	First degree	Journalism (broadcast)
	Male	20–25	Single	6 months	First degree	Advertising (agency)
Austrian	Male	21–25	Relationship with no child	2 years	Master degree	PR/Marketing (in-house)
participants	Male	21–25	Single with no child	1 year	Master degree	Social-Media-Marketing (agency)
	Female	31–35	Relationship with one child	10 years	First degree	Advertising (agency)
	Female	26–30	Relationship with no child	2 years	Master degree	PR/Marketing (in-house)
	Male	41–45	Married with one child	20 years	Master degree	Journalism (print + online)
	Female	26–30	Relationship with no child	3 years	Master degree	Journalism (print + online)
	Female	26–30	Single with no child	2 years	Master degree	Journalism (print + online)
	Female	36–40	Relationship with no child	13 years	Master degree	PR/Marketing (in-house)
	Female	46–50	Married with two children	14 years	Master degree	Journalism (print + online)
	Male	31–35	Married with two children	9 years	Master degree	Journalism (print + online)
	Female	26–30	Single with no child	2.5 years	Master degree	PR/Marketing (in-house)
	Male	26–30	Relationship with no child	2.5 years	Master degree	PR (in-house)

### Ethical considerations

To maintain high ethical standards, the protection of participants was treated as a central issue. Participation was voluntary, informed consent was obtained verbally, anonymity, and confidentiality were guaranteed (Berg & Lune, 2012; Eriksson & Kovalainen, 2008), and no incentives for participation were offered. The nature of the project was explained at the beginning of each interview; participants were informed that they would be asked to discuss their positions on certain questions with regard to work experiences and occupational stress. Interviewees were also informed that the data would be used for scientific and publication purposes. To protect participants’ anonymity and confidentiality, they were reassured that personal information and transcripts are kept and stored confidentially, that subjects remain nameless, and that any elements that might reveal interviewees’ identities were removed in the course of data analysis and reporting.

### Instrument and data collection

An interview guide with 11 questions was used in both countries. The list of predetermined questions included both open-ended and closed questions, which is feasible when doing semi-structured interviews (Eriksson & Kovalainen, 2008). The questions focused on the subjective experiences of employees with regard to demands and resources at work in the broader sense, and work-related ICT use in the narrower sense. In this article, ICT-related findings will be presented. Sample questions are: “Do ICTs improve your work efficiency? If yes, please explain in what way!” and “Do ICTs create more work strain for you? If yes, please explain in what way!” A review of the literature aided the authors in creating interview questions. The themes covered in the interviews were, therefore, predominantly generated from literature, and questions were developed to address ICTs as a possible source of work stress (e.g., Day et al., 2012), as well as to refer to several aspects of ICT use such as ubiquity, flexibility, and productivity improvements (e.g., Boswell & Olson-Buchanan, 2007; Day et al., 2012; Diaz et al., 2012; Fenner & Renn, 2010; Matusik & Mickel, 2011; O'Driscoll et al., 2010). The advantage of doing semi-structured interviews is that the predetermined outline of questions ensures that the set list of topics is covered in all interviews, yet remains open enough that the wording and order of questions may vary, and the interviewer may pursue topics initiated by the interviewee to probe beyond the answers to the predetermined questions (Berg & Lune, 2012; Eriksson & Kovalainen, 2008). This open feature of semi-structured interviews allowed the voice of the participants to flow into the study.

One of the authors conducted a first interview in Hong Kong to pretest the questions with regard to unambiguousness and comprehensibility. The predetermined questions turned out reasonable and comprehensible, and the interviewee did not mention any problems with the questions, the wording, or the process of the interview. Hence, no changes to the interview guide were deemed necessary after the pretest. Given that the interview guide was identical in the pretest and the main study, it was decided to add the pretest interview to the sample of the main study. The remaining 12 interviews in Hong Kong were conducted by a research assistant with a master's degree in communication. In Austria, all interviews were done by one of the authors. The duration of the interviews ranged from 21 to 66 min with an average of 31 min (Hong Kong: 33 min; Austria: 30 min) and were either conducted at participants’ work places or in public places such as cafés and restaurants, or via video telephony. Interviews were conducted in Cantonese or in Putonghua, depending on participants’ preferences, and in German, respectively. All interviews were audio recorded with the interviewees’ consent.

### Data analysis

The audio recordings were transcribed and translated into English. The researchers applied the procedure of inductive category development by Mayring (2000), which facilitated analysing the data in a way that allowed categories to emerge. Consistent with the theoretical foundation and the aim of this study, ICT stressors and ICT benefits in everyday work life, from an employee's perspective, served as selection criterion. The transcripts were worked through and processed based on this criterion, and categories were deduced step by step. With the help of NVivo so-called *nodes* were created within the software programme to organize and classify source data. A node is a collection of references about a specific theme or other area of interest. Nodes allow the gathering of related material in one place to look for emerging patterns and ideas. In the process of reading the transcripts, pieces of information were selected and coded either at a new or at an existing node. The nodes were revised, moved, merged, or renamed within feedback loops until the list of nodes had stabilized. To organize the source data more clearly, nodes were classified into *main nodes* and *subnodes* within NVivo. Specifically, ICT stressors and ICT benefits served as two overall main nodes, which are able to contain numerous subnodes, each which are able to contain their own subnodes. For example, remote access to information was divided into increased productivity, managing the information flow, and increased flexibility, and at the same time categorized as a subnode of instant accessibility, which in turn was a subnode of ICT benefits. This structure allowed us to recognize the categories that emerged most often and to link findings with each other. The results presented are the most frequent categories that emerged from the data.

## Results

### ICT stressors

All study participants agreed that ICTs are an additional source of work stress. Participants from Hong Kong reported that technologies, particularly wireless (converged) mobile devices, enable constant accessibility for colleagues, supervisors, or clients as the two following quotes exemplarily show:

My boss can check on me anytime. Once I travelled to Mainland China to write a story. It was already difficult to make contacts and conduct interviews. I had limited time for only one day and had to write the article at night. My boss kept asking me to find the core of the issue and dig out the manipulator behind the scene. He/she sat in the office in Hong Kong and typed these words on phone, but I was busy in asking questions on the spot. It was very annoying. (male participant, journalism, Hong Kong)

I have a client who contacts me every morning. He starts to work at 8:30 am, but I only start at 10:30 am. He gives me “morning calls” when he arrives at his office. He keeps sending me WhatsApp messages, asking me to respond as soon as possible. Every morning, when I turn off the alarm function on my mobile phone, I see his messages. He writes “call me back” every day. Even before the start of my working day, I get chased by my client. (male participant, advertising, Hong Kong)

These two quotes illustrate that employees are readily accessible via technologies when away from the office and outside of regular working hours. The first quote indicates that employees’ working processes can be repeatedly interrupted by text messages or emails, which can place additional pressures on employees as they may have to constantly shift their attention resulting in less focus on the actual task. Extending the finding by Sellberg and Susi (2014), whose study revealed that interruptions at the workplace through ICTs scatter a task throughout the day, this study indicates that technologies enable interruptions both in and out of the office. The second quote shows that technologies allow a constant connectivity to work outside of working hours, thus extending work hours and causing the boundaries between work and home life to blur. The participant describes a feeling of being chased before even starting his work day, indicating a feeling of stress and pressure. These quotes were categorized as *constant availability*.

Availability for work matters outside of working hours may make it increasingly difficult for employees to switch off and recover sufficiently from work demands. A diary study by Derks and Bakker (2012) showed that intensive work-related smartphone use during non-working hours increased work-related exhaustion due to the lack of psychological detachment, a state in which individuals mentally distance themselves from work (Sonnentag, 2012). The interviews indicate that ICTs may cause an increased response expectation, regardless of an individual's core working hours, which in turn can encourage constant availability. Two Austrian participants described:

When I get an email from my boss asking for information, I know that he expects me to answer right away, even if he doesn't explicitly mention it in the email. (female participant, PR & marketing, Austria)

At my work, there is no regulation, emails are being sent 24/7 and I am expected to reply at all times. I get phone calls at 8 pm or early in the morning. (female participant, journalism, Austria)

Similarly, Cousins and Robey (2015) found in their qualitative field studies among mobile workers, conducted in 2004 and 2008, that employees who are expected to be continuously available and responsive felt pressured to accept the blurring of boundaries between work and private life due to constant accessibility. In line with this, the interviews and other studies show that modern technologies may put employees under pressure to be almost always connected to work, which may result in increased stress (Day et al., 2010; Matusik & Mickel, 2011) and its adverse effects on employee health and well-being. Hence, ICTs may foster a certain pressure to stay connected to work outside of regular working hours. Therefore, this category of ICT stressors was labelled *connectivity pressure*.

The findings related to the overall aspect of availability from participants in both Austria and Hong Kong suggest that ICTs make employees available and accountable beyond the normal working hours, causing them to be constantly concerned with work matters and to be permanently connected to work, which may ultimately exhaust individuals’ resources. The question, however, is whether this pressure to be constantly available and connected is imposed from the outside or perhaps self-imposed. Although this aspect was not found among participants from Hong Kong, statements from Austrian participants strongly indicate that there may be an inner obligation to be available as the following quotes show:

I permanently check my emails, sometimes even in the middle of the night, I wake up and automatically reach to my phone, I feel pressured to do so. (female participant, journalism, Austria)

I got a smartphone from my employer, so I am always available, no matter when or where. I feel as if I have to be available at all times. For instance, when I'm in the car, I check my emails at every red traffic light. (female participant, PR & marketing, Austria)

The quotes indicate that employees may have high expectations of themselves concerning their own availability for work matters and thus creating self-imposed pressure to be constantly accessible. Especially, the first quote implies that the participant has work-related thoughts throughout the day, apparently even during the night, which may consequently prevent her from psychologically disengaging from work. The second quote further shows that the provision of wireless converged technology by the organization may also lead to and/or increase availability pressure. Employees may feel obligated to use the technology distributed to be connected to the workplace at all times, regardless of whether or not this is expected. Richardson and Benbunan-Fich (2011) reported similar findings and found that the distribution of wireless technology by the employer encourages individuals’ work connectivity behaviour outside of work hours. These quotes led to a category which we labelled *inner obligation for availability*.

A further but less significant theme that emerged from the data was higher workload due to ICT use. In this context, participants particularly referred to social media applications, most likely because these have extended the fields of work in the areas of advertising, public relations, and journalism and have become an integral part in these professions. These findings were categorized as *increased workload*. However, despite the additional workload caused by social networks, three participants also emphasized the benefits and described that social networks are an additional platform for being active, spreading stories, or generating awareness. The following quote exemplifies this paradox:

Using social media implies an additional time exposure and workload, but if my story spreads out and reaches more responses than it would have otherwise, the additional time and work were worth it. Effort and outcome have to be balanced. (male participant, journalism, Austria)

This quote indicates that if the benefits gained from ICTs outweigh the disadvantages, the increase in workload is not perceived as burdening, which illustrates well how the advantages of ICT use can be a benefit for employees, and as such, are able to buffer the impact of stressors.

Participants from Austria and Hong Kong both agreed that ICTs enable a constant availability, putting additional pressure on them, and possibly increasing the risk for experiencing a burnout. However, they differed in their descriptions. Participants from Hong Kong referred more strongly to a constant availability in connection with interruptions, whereas participants from Austria described a pressure to stay connected to work outside of working hours. In summary, results confirm the assumption that ICTs can be perceived as an occupational stressor. Particularly constant availability, connectivity pressure, an inner obligation for availability, and an increased workload emerged as major stressors of work-related ICT use.

### ICT benefits

All 13 interviewees in Hong Kong and the clear majority of the study participants in Austria agreed that ICTs help to improve work efficiency and to make work life easier. Hence, it can be assumed that ICTs can improve productivity. Nonetheless, one Austrian female participant disagreed and emphasized that ICTs do not improve work productivity, instead ICTs cause her to permanently feel stressed. All other participants were able to explain in one way or another how ICTs help to improve their work efficiency. For instance, study participants explained that ICTs help to enhance the internal flow of communication, to optimize the information exchange between employees, or to accelerate coordination processes with customers as the two following quotes illustrate:

Our company has a WhatsApp Group Chat with a dozen colleagues. This is a typical situation when I am in a press conference; once I receive the press release, I would take a picture of it and send it to my company. Or I would summarize it and make it into a 30-second news article. At the same time, the editor is able to revise the article at the office. This way, both colleagues inside and outside the office get an idea on what is going on at the press conference. (female participant, journalism, Hong Kong)

In the past when I created a design, the customer would come to the office, we would talk about the draft, and afterwards I would revise it if necessary. Nowadays the design is sent back and forth via email, everything goes a lot faster. (female participant, advertising, Austria)

These quotes illustrate that ICTs allow for more efficient communication and coordination processes both internally among employees and externally with customers, confirming previous scholars’ assumption that ICTs may facilitate communication and coordination, enabling better communication transfer, and allowing for increased information exchange (Day et al., 2010; Patel et al., 2012). In the first quote, the participant referred to WhatsApp, an instant messaging app. It is notable that although Austrian participants did not once refer to instant messaging apps, WhatsApp was an important issue among Hong Kong interviewees. WhatsApp was the most commonly mentioned electronic application by Hong Kong participants, representing a major ICT benefit for them. This may indicate that instant messaging apps may have already become an integral part of employees’ work life in the areas of advertising, public relations, and journalism in Hong Kong, but may have not yet found their way into these professions in Austria. Moreover, the following quote shows that WhatsApp enables quick and simplified exchange of (just-in-time) information and enhances communication:

People now are used to communicate via WhatsApp even in business. A typical situation, we can't find someone immediately via email. The person may have a conference, but he or she is able to check WhatsApp messages. We can still inform them immediately. This increases work efficiency and shortens the time of communication. (female participant, public relations, Hong Kong)

Therefore, these quotes were categorized as *improved communication processes*. These findings also indicate that improved communication processes are made possible by an immediate availability via wireless (converged) mobile devices, regardless of whether employees are in a meeting, at a press conference, or elsewhere outside the office. An important aspect that emerged in this context was the possibility of remote access to information. Participants reported finding it beneficial to access relevant business information independent of location and device as the following quote shows:

All my electronic devices are synchronized so that I can access my data anytime from any device. When I am in a meeting, for example, I can get all the relevant information I need by accessing the company's internal share point via my smartphone. This way, I always have all the information with me in digital form. (male participant, PR & marketing, Austria)

Participants further reported that this remote access via converged mobile devices helps them to better manage and control the flow of information. One participant described:

When I had an out-of-office appointment for a whole day in the past, I would have to go through hundreds of emails when coming back to the office before being able to do anything productive. This was more stressful than now, because now I can permanently work off my emails bit by bit throughout the day, no matter where I am. (male participant, journalism, Austria)

These findings led to a category of ICT benefits which we labelled *instant accessibility*. Mazmanian et al. (2006) reported similar findings in their qualitative study among employees in a small private equity firm; interviewees explained that frequently checking their emails via wireless email devices gives them a sense of control over the flow of information. A qualitative study among a police force in the UK discovered that constant access to information can actually help employees reduce the pressure created by information overload as information intake can be spread throughout the day (Allen & Shoard, 2005). Information overload is an especially relevant issue in connection with modern technologies because too much communication could lead to a flood of information (Day et al., 2010). This aspect was addressed by participants from both Austria and Hong Kong. Interviewees described how tedious and time-consuming it is to work through countless email (spam) messages and, in particular, emphasized the necessity of being able to differentiate between important and less important things.

Dealing with messages or information remotely, further, indicates that ICTs have made it easier and more feasible for employees to stay up to date and informed about ongoing work even when outside the office. This may consequently facilitate information processing and improve employees’ work ability. Accordingly, Austrian and Hong Kong participants (eight persons in total) reported that having location and device-independent access to information allows them to process or delegate tasks remotely, to answer customer queries while on the move, or to use dead time, such as time between meetings, more productively. Finally, accessibility regardless of time, location, and device allows a certain temporal and spatial flexibility in when or where to work (Diaz et al., 2012). This flexibility is also well described by the following quote:

I can check my e-mails when being away from the office, especially when I am waiting for something important. I don't have to sit at the desk throughout the day; this gives me a feeling of freedom. (female participant, PR & marketing, Austria).

Moreover, the findings indicate that ICTs may not only empower employees to organize their work in a reasonably flexible way, but may also support employees in balancing work and private life, thus positively contributing to work–family balance as the two following quotes illustrate:

For instance, if you have a dinner with friends at night, but you still have something to deal with. In the past you would have had to wait for the email [in the office], but now you can check the email and contact the client from everywhere via instant communication tools (female participant, public relations, Hong Kong).

If I don't get all the things done at work, I take my work home. I spend time with my family in the evening and then I continue working at 10 pm to finish my work. (male participant, journalism, Austria)

Hence, employees can use ICTs to their own personal advantage. Moreover, in contrast to previous literature, which noted that allowing work to creep into one's private life would promote work–family conflict (e.g., Diaz et al., 2012), that is, an interrole conflict in which the pressures from work and family roles are incompatible to some extent (Greenhaus & Allen, 2011; Greenhaus & Beutell, 1985; Greenhaus & Powell, 2003), the current findings suggest that the increased permeability as a result of the evolving technologies in the workplace can also support employees in better balancing work and private life, possibly improving employee well-being in the long term. Hence, consistently separating the private world from the world of work may not be the solution to diminish technology-induced stress. These findings were categorized as *increased flexibility*.

In summary, results confirm the notion that ICTs can be perceived as beneficial in working life. Participants reported profiting particularly from improved communication processes, instant accessibility to information independent of location and device, and increased flexibility in terms of working time and working place.

**Figure 1:**
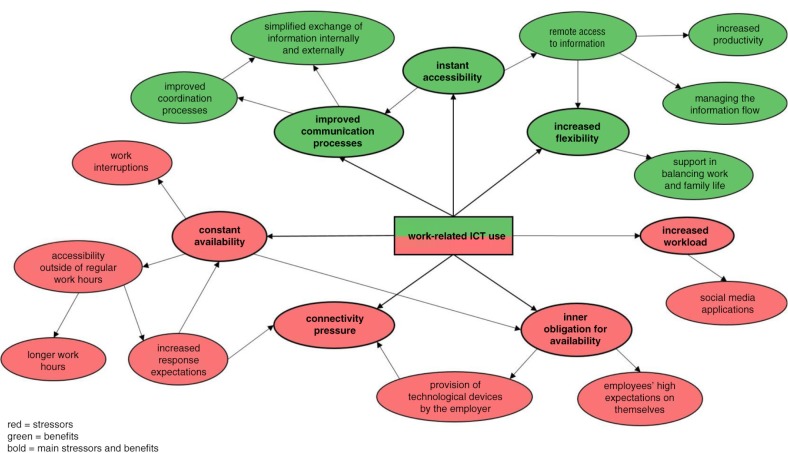
Benefits and stressors of work-related ICT use.

Figure 1 presents a summary and visualization of the main results.

## Discussion

This exploratory qualitative study examining ICT-related stressors and benefits on the job among employees in advertising, public relations, and journalism in Hong Kong and Austria confirmed the assumption that ICTs are perceived as both beneficial and detrimental, supporting the notion that ICTs constitute a double-edged sword (Diaz et al., 2012; Patel et al., 2012). Categories of stressors that emerged most often are constant availability, connectivity pressure, inner obligation for availability, and increased workload, whereas improved communication, instant accessibility, and increased flexibility constitute benefits as a result of technology use.

It is apparent that the aspect of availability, which emerged as a major theme from the data in both Hong Kong and Austria, is perceived simultaneously as a stressor and a benefit. ICTs allow an instant availability via wireless devices regardless of whether employees are in a meeting or at an out-of-office appointment, thus facilitating communication and coordination processes as well as information exchange. In Hong Kong, instant messaging apps have become an integral part of employees’ working life and have changed communication processes by enabling the quick and simplified exchange of just-in-time information. Furthermore, employees can access relevant business information independent of time, location, and device with the aid of converged mobile devices, which helps to better control the information flow, to use time more productively, and to stay informed about the ongoing work even outside the office. Beyond that, findings indicate that dealing with and accessing information remotely enables employees to organize their work with greater temporal and spatial flexibility. It should be noted that while previous studies had found that work-related ICT use during non-working hours is related to work–family conflict (Ayyagari, Grover, & Purvis, 2011; Diaz et al., 2012; Harris et al., 2011), which has been identified as a precursor to burnout (Allen, Herst, Bruck, & Sutton, 2000; Bacharach, Bamberger, & Conley, 1991; Demerouti, Bakker, & Schaufeli, 2005; Kinnunen, Vermulst, Gerris, & Mäkikangas, 2003; Peeters, Montgomery, Bakker, & Schaufeli, 2005), the current study extends this finding by indicating that the increasingly blurred boundaries between life domains as a result of the evolving technologies may actually create an opportunity for better balancing work and private life due to greater flexibility. These benefits help to improve employees’ work efficiency and work ability and may thus contribute to employee well-being.

However, being readily available inside and outside the office also means that employees are continuously accessible for colleagues, supervisors, or clients via technologies. In this context, the study's results suggest that ICTs enable constant interruptions of work processes in and out of the office, extend work beyond normal working hours, and increase expectations concerning response behaviour, all of which may increase work stress and place additional pressure on employees. Moreover, results showed that employees appear to be concerned with work matters more or less around the clock, checking messages in the morning right after getting up, or in the middle of the night. Consistent with previous findings, constant connectivity to work may ultimately foster an always-on mentality making it increasingly difficult for employees to disengage from work (Barber & Jenkins, 2014; Boswell & Olson-Buchanan, 2007; Mazmanian et al., 2006; Park et al., 2011). With regard to Hobfoll's (1989) Conservation of Resources Theory, failing to sufficiently recover from work can deplete individual's resources, ultimately increasing psychological stress, with exhaustion, a core component of burnout, as a potential outcome.

Although only noted among participants from Austria, availability pressure may be primarily attributed to an inner obligation rather than to an organizational expectation. Findings of this current study suggest that employees may have high expectations of themselves concerning their availability for work matters. Particularly the provision of technological devices by the employer may increase availability pressure, regardless of whether or not this is expected. Fenner and Renn (2010) found that an organizational climate that fosters the performance of extra-work behaviour at home with the help of technology may actually be interpreted by individuals as social pressure to exhibit this type of behaviour. Similarly, Richardson and Benbunan-Fich (2011) found that employees used communication technologies after hours for the purpose of work, especially if this type of behaviour was subjectively perceived as an organizational norm. Findings presented in this study likewise indicate that employees may feel obligated to be accessible at all times for colleagues, supervisors, or clients due to an unspoken and general expectation of constant availability via technological devices and applications. Thus, the pressure to be constantly available may indeed be self-imposed in response to individuals’ subjective perceptions of the behaviour, or expectations of important others in their organization, irrespective of whether or not permanent availability is explicitly expected by management. Considering that the fields of advertising, public relations, and journalism are highly competitive, the self-imposed pressure for constant availability may indeed be a crucial issue in the use of technologies promoting work stress and its consequences.

Employees from both Austria and Hong Kong reported that ICTs have made communication processes, the exchange of information, and the access to information, much easier, faster, and more effective. Hence, they help to increase employees’ work ability and productivity. Nevertheless, employers and employees should not overlook that evolving technologies in the workplace come with a cost. Particularly constant availability in and out of the office and beyond normal working hours constitutes an additional source of work stress. ICTs, thus, increase the risk of experiencing prolonged work stress, which may have adverse effects on employee health.

### Practical implications

The findings suggest that companies of the observed industries should raise their employees’ awareness of both benefits and stressors of ICT use. For example, internal communication measures can be used to promote a more responsible and balanced handling of technologies, which may ultimately help to increase and maintain employees’ well-being. Companies may want to develop and implement appropriate corporate directives concerning work-related ICT use with the goal of reducing technology-induced stress. They could also include this subject in their internal company communication, demonstrating that they are aware that technologies can be a significant stress factor in working life and actively address the topic of work stress and employee health. Considering that findings have indicated that the pressure to be constantly available may be a matter of individual choice, companies could make employees aware of their connectivity behaviour and explicitly declare organizational expectations regarding responsiveness during non-working hours. Given that findings have also shown that employees are readily and continuously accessible for colleagues and supervisors, it may be further beneficial to encourage employees to respect core working hours and to avoid sending emails or messages via instant messaging apps outside these times.

Implementing interventionist and preventative measures could help to reduce the risk of work-related stress while increasingly profiting from the benefits that arise from ICT use. Such measures are considered important because technology-related work stress may be a crucial issue in connection with stress in the work environment, psychological strain in the workplace, and overall employee health. Technobenefits can potentially contribute favourably to employees’ well-being, which is particularly important in light of the fact that taking health promoting measures related to positive outcomes (Svedberg, 2011) has become an important topic at the political, social, and economic level.

## Contributions, limitations, and directions for future research

The results should not be taken as an exhaustive examination of specific stressors and benefits resulting from work-related technology use, as the research design poses some limitations. All interviews had to be translated into English. The translation was conducted in a very thorough way, especially with regard to the key constructs. Nevertheless, there is always a risk of losing specific cultural or social connotations during translation. With regard to data analysis, the deductive interview approach may have influenced the inductive category development in such a way that the presumptions of the researchers might have crept in, which limits the methodological rigour of the analysis. Furthermore, it is acknowledged that the interview guide was pretested only in Hong Kong and that different interview technologies, that is, face-to-face and video telephony, were used. However, fast Internet connection enabled smooth interviews with no technical disturbances. Hence, interviews via video telephony were relatively comparable to face-to-face situations. Moreover, none of the participants that were involved in a video interview noted that this type of interview was unsuitable.

Despite these limitations, this study is, to the best of the authors’ knowledge, the first study to analyse technology use in the context of occupational stress among employees in Austria and Hong Kong. This study primarily contributes to the gap in research on benefits and downsides of work-related ICT use (Derks et al., 2014; Diaz et al., 2012). Its findings underpin the relevance of ICT use related to employee well-being and provide sufficient grounds for further exploration of the effect of ICT use on employee health and well-being. Given that research has found evidence of the effect of ICT on well-being (e.g., Day et al., 2012; Derks & Bakker, 2012), the study's results show specific aspects that deserve consideration in the relationship between ICT use and well-being. It would be particularly interesting to examine whether the effect of ICT use on employee well-being depends on whether ICT use is perceived more as an opportunity for better balancing work and private life or more as a cause for a conflict between life domains. This assumption is supported by the increasing awareness that burnout should no longer be treated as an exclusively work-related phenomenon but instead as a result of the complex interaction between work and non-work experiences (Grzywacz, Almeida, & McDonald, 2002; Lingard, 2004; Voydanoff, 2004). Hence, it is hypothesized that the perceived effect of ICT use on the reconciliation of work and private life (the perceived work-private life balance) moderates the effect of ICT use on employee health. Here a qualitative follow-up study may be fruitful to further examine under which conditions work-related ICT use causes a conflict, and under which it may promote a balance between work and private life. A quantitative study would allow for further analysis of the relationship between the variables and might yield interesting results. Testing this hypothesis would allow conclusions about preventative measures with regard to work-related ICT use that could support employees in balancing work and private life.

Another major finding was that accessibility pressure may arise due to an inner obligation of availability, which indicates that the effect of ICT use on well-being may depend on character traits in such a way that the effect depends on the extent to which employees feel they have to be constantly available for work matters. The self-imposed availability pressure also suggests that technological advances have influenced work culture and work identity to such an extent that being available may be interpreted as an indication of diligence and hard work. As the aspect of inner obligation for availability was only found among Austrian participants, future research may want to investigate the role that self-imposed pressure for constant availability plays in Hong Kong. One possible reason why Hong Kong participants did not mention this aspect may lie in their high work ethic and their belief in the relationship between hard work and performance, and performance and reward (Chiu & Kosinski, 1995; Chiu, Luk, & Tang, 2002; Lawler, 1973). Specifically, constant availability may be interpreted as hard-working, which might consequently result in a reward, such as merit pay or paid overtime, although this was not found in the current study. Given that cash-related compensations are identified as the most important factors to attract, retain, and motivate employees in Hong Kong (Chiu et al., 2002), they might accept being “always available” in exchange for monetary reward. It may be insightful to pursue the self-imposed pressure for availability in general, and the proposed assumption in particular, for example, in the form of a qualitative follow-up study among Hong Kong employees.

Future research might also look at possible sex differences in individual experiences related to ICT use in general, as well as in the perception of technology-induced stress in particular, considering that one female participant from Austria indicated feeling permanently stressed due to ICT use. It may also be of interest to examine whether and how generational differences exist in the perception of ICT use. In-depth interviews could show whether “digital natives,” who grew up with digital technologies surrounding them (Prensky, 2001), differ in their perception of technology-related stressors and benefits as compared to “digital immigrants,” who grew up in an environment without computers and the Internet (Prensky, 2001). In addition, other socio-demographic factors like education or whether an employee has children, as well as the age of the children, might influence the perception of ICTs as stressors or benefits and the perceived work stress. Examining the influence of sex, age, or other socio-demographic factors on experiences related to ICT use could also be useful in the development of ICT-related interventions on an organizational level to diminish technology-induced stress. A qualitative study, for example, in the form of focus groups, could reveal whether different measures should be taken depending on employees’ socio-demographic characteristics.

Another possible future research avenue could be to extend the study to areas beyond advertising, public relations, and journalism and to extend it to other countries. Future studies may also conduct quantitative studies to develop a model of how technology-related benefits and stressors impact employee well-being that can be tested with inferential statistical analyses.

Following these considerations, further research in this field could help improve and maintain employees’ health and well-being and diminish the risk of health impairments in the long term, thus reducing the costs associated with absenteeism due to stressed, exhausted, burnt-out employees.
